# Association Between Surgery Preference and Receipt in Ductal Carcinoma In Situ After Breast Magnetic Resonance Imaging: An Ancillary Study of the ECOG-ACRIN Cancer Research Group (E4112)

**DOI:** 10.1001/jamanetworkopen.2022.10331

**Published:** 2022-05-10

**Authors:** Soudabeh Fazeli, Bradley S. Snyder, Ilana F. Gareen, Constance D. Lehman, Seema A. Khan, Justin Romanoff, Constantine A. Gatsonis, Ralph L. Corsetti, Habib Rahbar, Derrick W. Spell, Kenneth B. Blankstein, Linda K. Han, Jennifer L. Sabol, John R. Bumberry, Kathy D. Miller, Joseph A. Sparano, Christopher E. Comstock, Lynne I. Wagner, Ruth C. Carlos

**Affiliations:** 1 Department of Radiology, University of California San Diego, San Diego; 2 Center for Statistical Sciences, Brown University School of Public Health, Providence, Rhode Island; 3 Department of Epidemiology, Brown University School of Public Health, Providence, Rhode Island; 4 Department of Radiology, Massachusetts General Hospital, Harvard Medical School, Boston; 5 Department of Medicine, Northwestern University Feinberg School of Medicine, Chicago, Illinois; 6 Department of Surgery, Tulane University School of Medicine, New Orleans, Louisiana; 7 Department of Radiology, University of Washington, Seattle; 8 Gulf South NCORP, New Orleans, Louisiana; 9 Hunterdon Regional Cancer Center, Flemington, New Jersey; 10 Department of Surgery, Indiana University, Indianapolis; 11 Department of Surgery, Lankenau Medical Center, Wynnewood, Pennsylvania; 12 Department of Surgery, Mercy Hospital Springfield, Springfield, Missouri; 13 Indiana University Melvin and Bren Simon Comprehensive Cancer Center, Indianapolis; 14 Department of Hematology-Oncology, Mount Sinai Health System, New York, New York; 15 Department of Radiology, Memorial Sloan-Kettering Cancer Center, New York, New York; 16 Wake Forest School of Medicine, Wake Forest Baptist Comprehensive Cancer Center, Winston Salem, North Carolina; 17 Department of Radiology, University of Michigan, Ann Arbor; 18 Program for Women’s Health Effectiveness Research, University of Michigan, Ann Arbor; 19 Institute for Health Policy and Innovation, University of Michigan, Ann Arbor

## Abstract

**Importance:**

Guiding treatment decisions for women with ductal carcinoma in situ (DCIS) requires understanding patient preferences and the influence of preoperative magnetic resonance imaging (MRI) and surgeon recommendation.

**Objective:**

To identify factors associated with surgery preference and surgery receipt among a prospective cohort of women with newly diagnosed DCIS.

**Design, Setting, and Participants:**

A prospective cohort study was conducted at 75 participating institutions, including community practices and academic centers, across the US between March 25, 2015, and April 27, 2016. Data were analyzed from August 2 to September 24, 2021. This was an ancillary study of the ECOG-ACRIN Cancer Research Group (E4112). Women with recently diagnosed unilateral DCIS who were eligible for wide local excision and had a diagnostic mammogram within 3 months of study registration were included. Participants who had documented surgery and completed the baseline patient-reported outcome questionnaires were included in this substudy.

**Exposures:**

Women received preoperative MRI and surgeon consultation and then underwent wide local excision or mastectomy. Participants will be followed up for recurrence and overall survival for 10 years from the date of surgery.

**Main Outcomes and Measures:**

Patient-reported outcome questionnaires assessed treatment goals and concerns and surgery preference before MRI and after MRI and surgeon consultation.

**Results:**

Of the 368 participants enrolled 316 (86%) were included in this substudy (median [range] age, 59.5 [34-87] years; 45 women [14%] were Black; 245 [78%] were White; and 26 [8%] were of other race). Pre-MRI, age (odds ratio [OR] per 5-year increment, 0.45; 95% CI, 0.26-0.80; *P* = .007) and the importance of keeping one’s breast (OR, 0.48; 95% CI, 0.31-0.72; *P* < .001) vs removal of the breast for peace of mind (OR, 1.35; 95% CI, 1.04-1.76; *P* = .03) were associated with surgery preference for mastectomy. After MRI and surgeon consultation, MRI upstaging (48 of 316 [15%]) was associated with patient preference for mastectomy (OR, 8.09; 95% CI, 2.51-26.06; *P* < .001). The 2 variables with the highest ORs for initial receipt of mastectomy were MRI upstaging (OR, 12.08; 95% CI, 4.34-33.61; *P* < .001) and surgeon recommendation (OR, 4.85; 95% CI, 1.99-11.83; *P* < .001).

**Conclusions and Relevance:**

In this cohort study, change in patient preference for DCIS surgery and surgery received were responsive to MRI results and surgeon recommendation. These data highlight the importance of ensuring adequate information and ongoing communication about the clinical significance of MRI findings and the benefits and risks of available treatment options.

## Key Points

**Question:**

What factors are associated with surgery preference (wide local excision vs mastectomy) among a prospective cohort of women with newly diagnosed ductal carcinoma in situ (DCIS) before and after magnetic resonance imaging (MRI) and surgeon recommendation?

**Findings:**

In this cohort study of 368 women diagnosed with DCIS, surgical preference was statistically significantly associated with age and treatment goals and concerns before MRI receipt. Magnetic resonance imaging upstaging, surgeon recommendation, the self-reported importance of keeping one's breast, and cancer worry were associated with received surgery.

**Meaning:**

These findings suggest that surgery preference for women with DCIS may respond dynamically to additional diagnostic information conferred by MRI and surgeon recommendation.

## Teaser

This cohort study examines the preferences of women for treatment of ductal carcinoma in situ on the basis of magnetic resonance imaging findings and surgeon recommendations.

## Introduction

Ductal carcinoma in situ (DCIS) accounted for more than 48 000 new breast cancer diagnoses in 2019, representing almost 20% of all breast cancer diagnoses in the US.^1^ The incidence of DCIS has increased with the implementation of routine screening mammography.^2^ Ductal carcinoma in situ encompasses a spectrum of preinvasive lesions originating within healthy breast tissue, with variable natural history and risk of progression to invasive breast cancer.^3^ This spectrum heterogeneity contributes to heterogeneous surgical management including wide local excision (WLE), with or without radiotherapy, and mastectomy, with similar reported survival rates.^4^ Given the large number of DCIS treatment options, patient preference is an important component in selecting one option over another.^5^ Lehman et al^6^ evaluated the diagnostic contribution of breast MRI findings in individuals with newly diagnosed DCIS who were eligible for WLE, describing the frequency of surgery received. Additional diagnostic information, including from MRI, can further personalize treatment decisions.

Research on treatment preference, goals, and concerns has been conducted primarily among patients with invasive breast cancer. These patients vary in how much they value treatment elements, including decreasing cancer recurrence, eliminating the need for radiotherapy, and effects on postoperative body image, when making decisions about breast surgery.^7,8,9^ Despite the added complexity of decision-making in DCIS, similar data on surgery preference are lacking, with outcomes associated with preoperative MRI and surgeon recommendation as mediators of surgery preference remaining unknown. Therefore, among a prospective cohort of women with newly diagnosed DCIS receiving MRI for surgery planning, the aims of this study were to (1) assess women’s preference for WLE vs mastectomy before and after MRI and surgeon recommendation, (2) report concordance between surgery preference and initial surgery received, and (3) assess factors associated with surgery preference and initial surgery received.

## Methods

The study was approved by the National Cancer Institute, Division of Cancer Prevention, and by the local institutional review board at each participating site, as well as by the Brown University Institutional Review Board. Written informed consent was obtained from all participants. Reporting of this study follows the Strengthening of the Reporting of Observational Studies in Epidemiology (STROBE) reporting guideline for cohort studies.

### Data and Sample

This cohort study was conducted at 75 participating institutions, including community practices and academic centers, as an ancillary study to a prospective nonrandomized clinical trial coordinated by the ECOG-ACRIN Cancer Research Group (E4112). Enrollment dates were between March 25, 2015, and April 27, 2016. Primary trial results and details on study design and participating institutions have been described.^6^ Study collection of demographic variables, including self-identified race and ethnicity, was required by the National Cancer Institute, Division of Cancer Prevention; however, participants were not required to respond to any of the demographic questions. Data analysis was performed from August 2 to September 24, 2021.

In brief, to be eligible for the parent study, participants had unilateral DCIS, were candidates for WLE, and received a diagnostic mammogram of the affected breast no more than 3 months before trial registration. Once enrolled, participants underwent a breast MRI and additional imaging and/or biopsy if considered to be indicated by the MRI findings. Following a preoperative surgical consultation, women underwent WLE or mastectomy. Patients for whom the initial attempted WLE was not successful (defined as tumor-free surgical margin of <2 mm or evidence of microinvasive or invasive carcinoma) underwent further WLEs or mastectomy. Clinical and patient-reported outcomes (PROs) were scheduled to be collected at the time of registration (time point T0) and after MRI and surgeon consultation and before surgery (time point T1) (eFigure 1 in the Supplement). Surgeon recommendation was scheduled to be collected before surgery.

### Outcomes

#### Surgery Preference, Concordance With Surgery Received, and Reexcision Frequency

Participants’ surgery preferences were examined at T0 and T1 using a single item (Which treatment is your personal preference to treat your DCIS?) with 3 response categories (lumpectomy, mastectomy, or I don’t know) adapted from the Breast Cancer Surgery Decision Quality Instrument by Sepucha et al.^9,10^ Surgery received was assessed after completion of all surgeries (WLE, mastectomy, or attempted WLE followed by mastectomy). Concordance between surgery preference and surgery receipt was defined as the woman receiving the type of surgery that matched her preferred surgery choice at T1. Women who preferred WLE and had WLE as the first procedure were counted as concordant, regardless of the final surgery.

#### Factors Assessed for Association With Outcomes

Demographic characteristics included age, self-reported race, insurance status, and Area Deprivation Index (ADI).^11,12^ The ADI allows for rankings of neighborhoods by socioeconomic disadvantage and includes factors for the theoretical domains of income, educational level, employment, and housing quality.^12^ The ADI ranks 1 as the lowest and 100 as the highest levels of disadvantage within the US.

Cancer worry was measured at T0 using the 3-item cancer worry subscale from the Assessment of Survivor Concerns.^13^ Each item has a 4-category response scale of 1 (not at all), 2 (a little bit), 3 (somewhat), and 4 (very much). The mean of the 3 cancer worry items (fear of cancer recurrence, new cancer diagnosis, and diagnostic tests) was determined for each participant, arriving at a semicontinuous measure ranging from 1 to 4, with higher values indicating greater levels of cancer worry.

Women’s treatment goals and concerns were examined at T0 using 8 items (Table 1) adapted from Sepucha et al^9,10^ and Hawley et al^14^ to assess the importance of the following domains in surgical decision-making: cancer recurrence reduction, risk of reoperation due to incomplete margins, need for radiotherapy, and postsurgical body image and sexual function. Each response item was measured using an 11-point scale, ranging from 0 (not at all important) to 10 (extremely important).

**Table 1.** Patient Demographic and Clinical Characteristics and PRO Data

Variable	Eligible with MRI performed and known final surgery status (n = 339)	T0 PRO completed (n = 316)	T0 and T1 PRO completed (n = 250)
Age, median (range), y	60 (34-87)	59.5 (34-87)	60 (34-87)
Race, No. (%)			
Black	49 (14)	45 (14)	29 (12)
White	262 (77)	245 (78)	202 (81)
Other^a^	28 (8)	26 (8)	19 (8)
Insurance status, No. (%)			
Private	261 (77)	244 (77)	191 (76)
Medicare/other government insurance	62 (18)	59 (19)	51 (20)
Medicaid/uninsured	16 (5)	13 (4)	8 (3)
Area Deprivation Index, median (IQR)^b^	44 (27-65)	44 (27-65)	44 (27-63)
MRI upstaging, No. (%)	53 (16)	48 (15)	39 (16)
Surgeon recommendation of mastectomy, No. (%)	72 (21)	70 (22)	57 (23)
ASC cancer worry subscale score, median (IQR)^c^	NA	2.3 (2.0-3.0)	2.0 (1.7-3.0)
Treatment goals and concerns, median (IQR)^c^^,^^d^	NA		
How important is it to you to keep your breast?	NA	7 (5-10)	7 (5-10)
How important is it to you to remove your entire breast to gain peace of mind?	NA	5 (2-8)	5 (2-8)
How important is it to you to avoid having radiation?	NA	6 (5-9)	5 (4-8)
How important is it to you to avoid the possibility of a second surgery to remove more cancer?	NA	9 (7-10)	9 (5-10)
How important is it to you to reduce the chances of the cancer coming back?	NA	10 (10-10)	10 (10-10)
How important is it that the type of surgery you have would not interfere with your sex life in the long term?	NA	5 (0-8)	5 (0-8)
How important is it that the type of surgery you have would not make you feel bad about your body, like you were disfigured?	NA	7 (3-9)	7 (3-9)
How important is it that the type of surgery you have would allow you to feel feminine?	NA	7 (4-9)	7 (5-9)

Abbreviations: ASC, Assessment of Survivor Concerns; MRI, magnetic resonance imaging; NA, not applicable; PRO, patient-reported outcomes; T0, time of registration; T1, time after MRI and surgeon consultation and before surgery.

^a^ Includes American Indian/Alaskan Native, Asian, multiple races, not reported, and unknown.

^b^ An Area Deprivation Index rank of 1 indicates the lowest level of disadvantage within the nation; an Area Deprivation Index rank of 100 indicates the highest level of disadvantage.

^c^ Solicited at time point T0.

^d^ Each treatment goal was solicited using an 11-point scale, ranging from 0 (not at all important) to 10 (extremely important).

We assessed clinical factors, including radiologist assessment of upstaging by MRI and surgeon recommendation. Upstaging by MRI was based on the opinion of the interpreting radiologist that the MRI demonstrated cancer extent that would influence the patient to select mastectomy, based either on additional foci of disease or the primary DCIS tumor being too large for WLE. Additional information on data collection is included in the eMethods in the Supplement.

### Statistical Analysis

The frequency of preference for WLE vs mastectomy at T0, proportion of women who changed their surgery preference between T0 and T1, and those who received surgery concordant with their indicated surgery preference at T1 were calculated. In addition, descriptive statistics were generated to characterize the cohort, including sociodemographic and PRO data at baseline.

Univariable and multivariable logistic regression models were fit to examine potential associations between sociodemographic and baseline PRO data and surgery preference and surgery receipt. Separate models were fit for surgery preference at T0, surgery preference at T1, and initial surgery received (WLE/attempted WLE vs mastectomy). Sociodemographic covariates included age, race, insurance status, and ADI rank. Self-reported ethnicity data were collected separately from self-reported race data but were omitted from analysis due to limitations on the number of covariates that could be included in the statistical models. Baseline PRO covariates included the ASC cancer worry subscale and women’s treatment goals and concerns. For treatment goals and concerns (Table 1), only 4 of 8 items (items 1-3 and 6) were included in the regression analyses; the remaining items were excluded owing to small variability in responses (items 4 and 5) or high degree of similarity/collinearity with other items (items 7 and 8). Models for surgery preference at T1 and initial surgery received further included MRI upstaging and surgeon recommendation as additional covariates. For surgery preference at T0 and T1, women with uncertain preference were excluded (T0: 16%; T1: 3%), and an ordinary logistic regression model was fit using a binary outcome (WLE vs mastectomy). However, given the larger proportion of women with uncertain preference at T0, a second multinomial logistic regression model was also fit, using a 3-category outcome (WLE vs mastectomy vs I don’t know).

Owing to the small number of women with preference for mastectomy, especially at T0, estimation of all logistic regression models was performed using the penalized maximum likelihood method proposed by Firth.^15^ This method has been shown to reduce small-sample bias in ordinary maximum likelihood estimation and can also help overcome issues of separation.^16^ The discriminatory ability of each model was assessed using the C statistic. Internal validation was conducted using bootstrap resampling.^17^ Both the apparent performance and the optimism-corrected performance are reported.

A classification (or decision) tree was constructed to allow a simpler visual presentation of factors associated with the initial surgery received.^18^ The same covariates were entertained as for the logistic regression model. The tree was pruned using 10-fold cross-validation, with nodal impurity assessed using the Gini index. Missing responses were assigned to the most popular (largest) node. Internal validation (via 10-fold cross-validation) was used to assess the misclassification rate. Because a small number of ADI rankings were based on post office boxes or only the 5-digit zip code, a sensitivity analysis was conducted removing these patients to assess study findings.

Data were analyzed using SAS, version 9.4 software (SAS Institute Inc) and R, version 4.0.4 software (R Foundation for Statistical Analysis). All reported *P* values are 2-sided, with the significance threshold set to .05. Results of complete case analyses are reported. Because the aims of this ancillary study constituted secondary and tertiary aims of the primary trial protocol, analyses were considered hypothesis-generating, and so adjustment for multiplicity of inference was not conducted.

## Results

### Participant Characteristics

**Figure 1.**
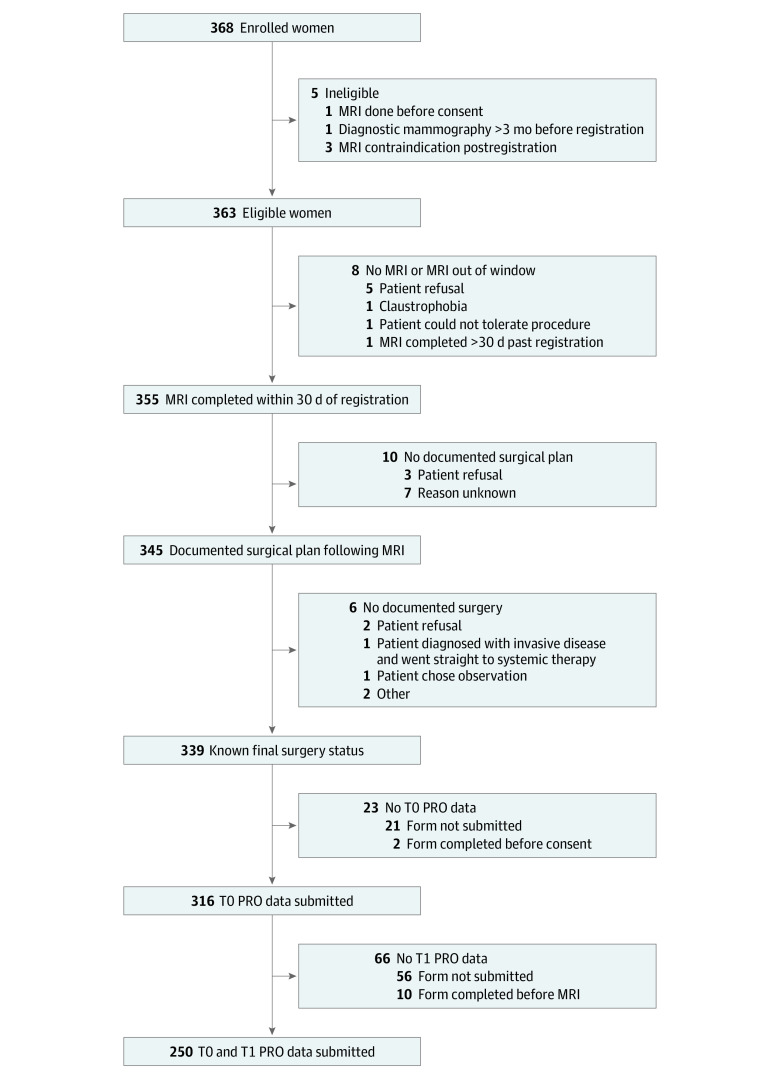
Study Flow Diagram MRI indicates magnetic resonance imaging; PRO, patient-reported outcomes; T0, time of registration; T1, time after MRI and surgeon consultation and before surgery.

Of the 368 women enrolled into E4112, 355 (96%) completed the study MRI and 339 (92%) had documented surgery with known final status (Figure 1). Of these 339 women, 316 (93%) completed the T0 questionnaire and 250 (74%) completed both the T0 and T1 questionnaires. Median age of the women was 59.5 (range, 34-87) years. Forty-five (14%) of the women were Black, 245 (78%) were White, and 26 (8%) were of other racial groups (American Indian/Alaskan Native, Asian, multiple races, not reported, and unknown). Other sociodemographic characteristics, clinical data, and PRO responses are summarized in Table 1.

Women varied in their treatment goals and concerns (Table 1). Overall, reducing the chances of cancer recurrence had the highest importance and the least variability on the 11-point scale among women at T0 (median, 10; IQR,10-10). Avoiding the possibility of a second surgery to remove more cancer (median, 9; IQR, 7-10), keeping the breast (median, 7; IQR, 5-10), and body image concerns (median, 7; IQR, 3-9) were also important considerations, although these elements exhibited greater variability and wider response distributions.

### Surgery Preference

**Figure 2.**
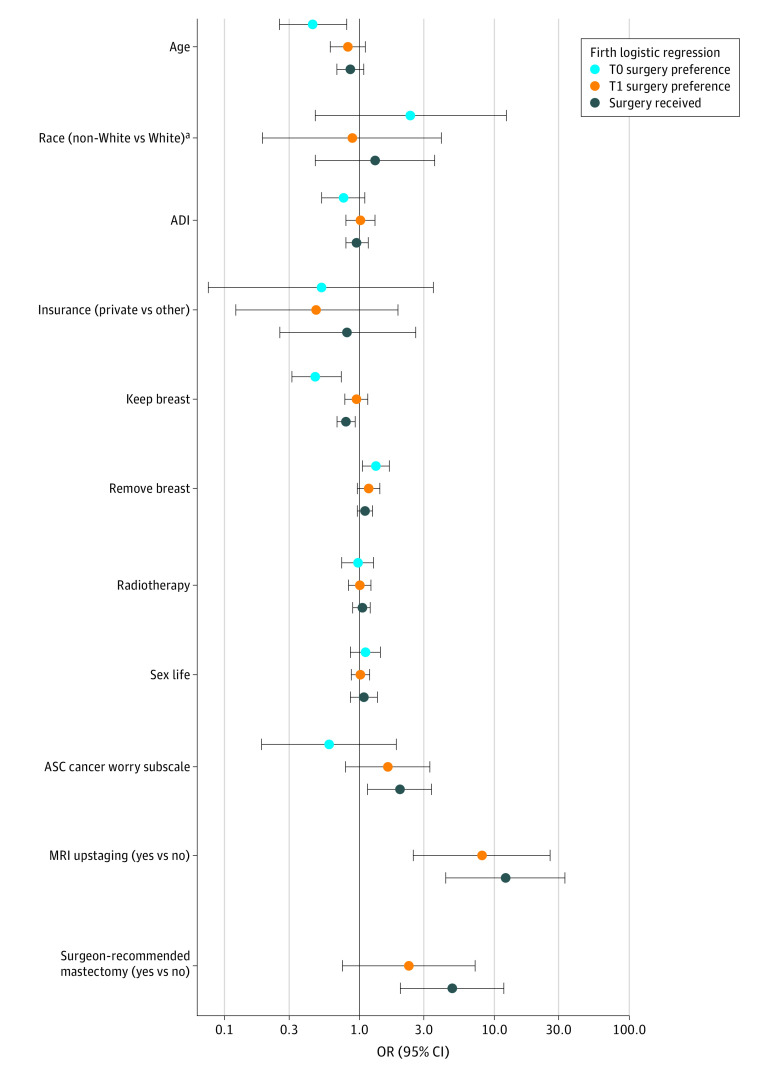
Adjusted Odds Ratios (ORs) From Multivariable Logistic Regression Models for Surgery Preference at Time of Registration (T0), Surgery Preference After Magnetic Resonance Imaging (MRI) and Surgeon Consultation and Before Surgery (T1), and Initial Surgery Received Firth penalized maximum likelihood logistic regression models were used. The x-axis is on the log scale, where odds ratios greater than 1 favor mastectomy. For categorical variables, the OR is interpreted in association with the indicated reference level. For age, the OR is interpreted per 5-year increase; for area deprivation index (ADI), the OR is interpreted per 10-percentile increase; for patient-reported outcome (PRO) variables modeled as continuous covariates (keep breast, remove breast, radiation, sex life, Assessment of Survivor Concerns [ASC] cancer worry subscale), the OR is interpreted per 1-unit increase on the respective scale. ^a^For race (non-White vs White), there were limitations as to the number of covariates that could be included in the statistical models based on the smaller number of women who preferred mastectomy, and this includes the number of categories for categorical covariates. Given that, race was conceived as binary as White (the predominant racial category) versus all other categories. All other categories include American Indian/Alaskan Native, Asian, Black, multiple races, not reported, and unknown.

At T0, of the 316 women, 80% (252 of 316) preferred WLE, 3% (11 of 316) preferred mastectomy, and 16% (50 of 316) were unsure of their surgery preference, with 3 missing responses. We initially conducted multivariable logistic regression analysis after excluding women who were unsure of their surgery preference (Table 2; Figure 2). The only sociodemographic characteristic significantly associated with preference for mastectomy was age (odds ratio [OR] per 5-year increase, 0.45; 95% CI, 0.26-0.80; *P* = .007), with older women less likely to prefer mastectomy. The importance of keeping one’s breast was associated with decreased odds (OR, 0.48; 95% CI, 0.31-0.72; *P* < .001), and the importance of removing the entire breast for peace of mind was associated with increased odds of preferring mastectomy (OR, 1.35; 95% CI, 1.04-1.76; *P* = .03). Unadjusted (univariable) estimates for all models can be found in eTable 1 in the Supplement.

**Table 2.** Multivariable Firth Penalized Maximum Likelihood Logistic Regression Models for Surgery Preference at T0, Surgery Preference at T1, and Initial Surgery Received^a^

Independent variables	T0 surgery preference		T1 surgery preference		Initial surgery received	
	Adjusted OR (95% CI)^b^	*P* value	Adjusted OR (95% CI)^b^	*P* value	Adjusted OR (95% CI)^c^	*P* value
Age (continuous, per 5-y increment)	0.45 (0.26-0.80)	.007	0.82 (0.61-1.11)	.20	0.86 (0.67-1.09)	.20
Race: non-White vs White^d^	2.40 (0.47-12.28)	.29	0.88 (0.19-4.06)	.87	1.30 (0.47-3.63)	.61
Insurance status: private vs Medicare/Medicaid/other government insurance/uninsured	0.52 (0.08-3.63)	.51	0.48 (0.12-1.93)	.30	0.82 (0.25-2.63)	.73
ADI (continuous, per 10-percentile increase)	0.76 (0.52-1.10)	.14	1.02 (0.79-1.31)	.91	0.97 (0.79-1.18)	.73
Treatment goals and concerns						
How important is it to you to keep your breast? (continuous)	0.48 (0.31-0.72)	<.001	0.95 (0.78-1.15)	.58	0.80 (0.68-0.93)	.004
How important is it to you to remove your entire breast to gain peace of mind? (continuous)	1.35 (1.04-1.76)	.03	1.17 (0.97-1.43)	.10	1.10 (0.97-1.26)	.15
How important is it to you to avoid having radiation? (continuous)	0.97 (0.74-1.27)	.83	1.01 (0.83-1.22)	.95	1.04 (0.89-1.21)	.61
How important is it that the type of surgery you have would not interfere with your sex life in the long term? (continuous)	1.11 (0.86-1.43)	.43	1.02 (0.87-1.19)	.84	1.08 (0.93-1.24)	.32
ASC cancer worry subscale score (continuous)	0.59 (0.19-1.87)	.37	1.63 (0.79-3.36)	.18	1.98 (1.14-3.43)	.02
MRI upstaging: yes vs no	NA	NA	8.09 (2.51-26.06)	<.001	12.08 (4.34-33.61)	<.001
Surgeon recommended mastectomy: yes vs no	NA	NA	2.33 (0.75-7.25)	.15	4.85 (1.99-11.83)	<.001
Apparent *C* statistic	0.97 (0.95-1.00)		0.86 (0.75-0.98)		0.91 (0.86-0.96)	
Optimism-corrected *C* statistic	0.93		0.79		0.88	

Abbreviations: ADI, Area Deprivation Index; ASC, Assessment of Survivor Concerns; NA, not applicable; OR, odds ratio; T0, time of registration; T1, time after MRI and surgeon consultation and before surgery; WLE, wide local excision.

^a^ For the surgery preference models, women who were unsure of their preference were excluded (n = 50 at T0, and n = 8 at T1).

^b^ Probability of mastectomy preference was modeled. Thus, an adjusted OR greater than 1 indicates higher odds of preferring mastectomy compared with WLE. For categorical variables, the OR is interpreted in relation to the indicated reference level. For age, the OR is interpreted per 5-year increase; for ADI, the OR is interpreted per 10-percentile increase; for patient-reported outcome variables modeled as continuous covariates (keep breast, remove breast, avoid radiation, sex life, ASC cancer worry subscale), the OR is interpreted per 1-unit increase on the respective scale.

^c^ Probability of mastectomy was modeled. Thus, an adjusted OR greater than 1 indicates higher odds of the patient receiving mastectomy vs WLE. Odds ratios are interpreted as explained in footnote b.

^d^ There were limitations as to the number of covariates that could be included in the statistical models based on the smaller number of women who preferred mastectomy, and this includes the number of categories for categorical covariates. Given that, race was conceived as binary as White (the predominant racial category) versus all other categories. All other categories include American Indian/Alaskan Native, Asian, Black, multiple races, not reported, and unknown.

Given that 16% of women were unsure of their surgery preference at study entry (T0), we also conducted multinomial logistic regression analysis, explicitly modeling the response as a 3-category outcome: preference for WLE, preference for mastectomy, or unsure (eTable 2 in the Supplement). The same variables were associated with preference for mastectomy vs WLE as in the previous model, with similar point estimates: age (OR per 5-year increase, 0.59; 95% CI, 0.39-0.90; *P* = .01), importance of keeping one’s breast (OR, 0.59; 95% CI, 0.45-0.76; *P* < .001), and removing the entire breast for peace of mind (OR, 1.43; 95% CI, 1.10-1.84; *P* = .006). Furthermore, using this model, we assessed surgery preference uncertainty compared with preference for WLE or mastectomy: older women were more likely to be uncertain than to choose mastectomy (OR per 5-year increase, 1.77; 95% CI, 1.15-2.72; *P* = .009), women who valued keeping their breast were less likely to be uncertain than to choose WLE (OR, 0.69; 95% CI, 0.61-0.79; *P* < .001), and those who valued removing their entire breast to gain peace of mind were more likely to be uncertain than to choose WLE (OR, 1.23; 95% CI, 1.10-1.39; *P* < .001).

Among 250 women who completed PRO questionnaires at both T0 and T1, where the T1 questionnaire was completed after MRI and surgeon consultation, 88% (219 of 250) of women preferred WLE, 9% (22 of 250) preferred mastectomy, and 3% (8 of 250) were unsure of their surgery choice, with 1 missing response. We conducted multivariable logistic regression similar to the analysis at time point T0, with MRI upstaging and surgeon recommendation included as additional variables (Table 2, Figure 2). Magnetic resonance imaging upstaging represented the only significant factor in surgery preference at T1 (OR, 8.09; 95% CI, 2.51-26.06; *P* < .001).

Between T0 and T1, 48 of 250 women (19%) changed their surgery preference. Women were not queried as to the reason for change in preference. However, 13 women changed from WLE to mastectomy; of these 13, 8 (62%) had MRI upstaging, and the same percentage had a surgeon recommendation of mastectomy. Four women changed preference from mastectomy to WLE; none had MRI upstaging or surgeon recommendation of mastectomy (eFigure 2 in the Supplement).

### Surgery Received

Among this cohort of women with DCIS, 84% (285 of 339) received WLE or attempted WLE. Furthermore, 79% of the women (269 of 339) underwent only 1 surgery (WLE or mastectomy) and 21% (70 of 339) underwent multiple surgeries (maximum, 3). Of 285 women initially undergoing attempted WLE, 11 (4%) ultimately received mastectomy.

Among women with known final surgery status and baseline PRO data, 15% (48 of 316) had upstaging by MRI. A higher percentage of women with MRI upstaging received a mastectomy (27 of 48 [56%]) compared with women without MRI upstaging (19 of 261 [7%]); 2% (7 of 316) had missing MRI upstaging status. This association was also found in a multivariable logistic regression model predicting initial surgery (Table 2, Figure 2), in which the adjusted odds of receiving mastectomy were higher in women with MRI upstaging (OR, 12.08; 95% CI, 4.34-33.61; *P* < .001). In addition, women with a surgeon recommendation of mastectomy (OR, 4.85; 95% CI, 1.99-11.83; *P* < .001) were more likely to receive mastectomy, women who valued keeping their breast were less likely to receive mastectomy (OR, 0.80; 95% CI, 0.68-0.93; *P* = .004), and the odds of receiving mastectomy increased with increasing cancer worry (OR, 1.98; 95% CI, 1.14-3.43; *P* = .02). The optimism-corrected C statistic for this model based on internal validation was 0.88, indicating that the combined covariates yielded excellent discrimination between women who received WLE or attempted WLE vs mastectomy (Table 2).

**Figure 3.**
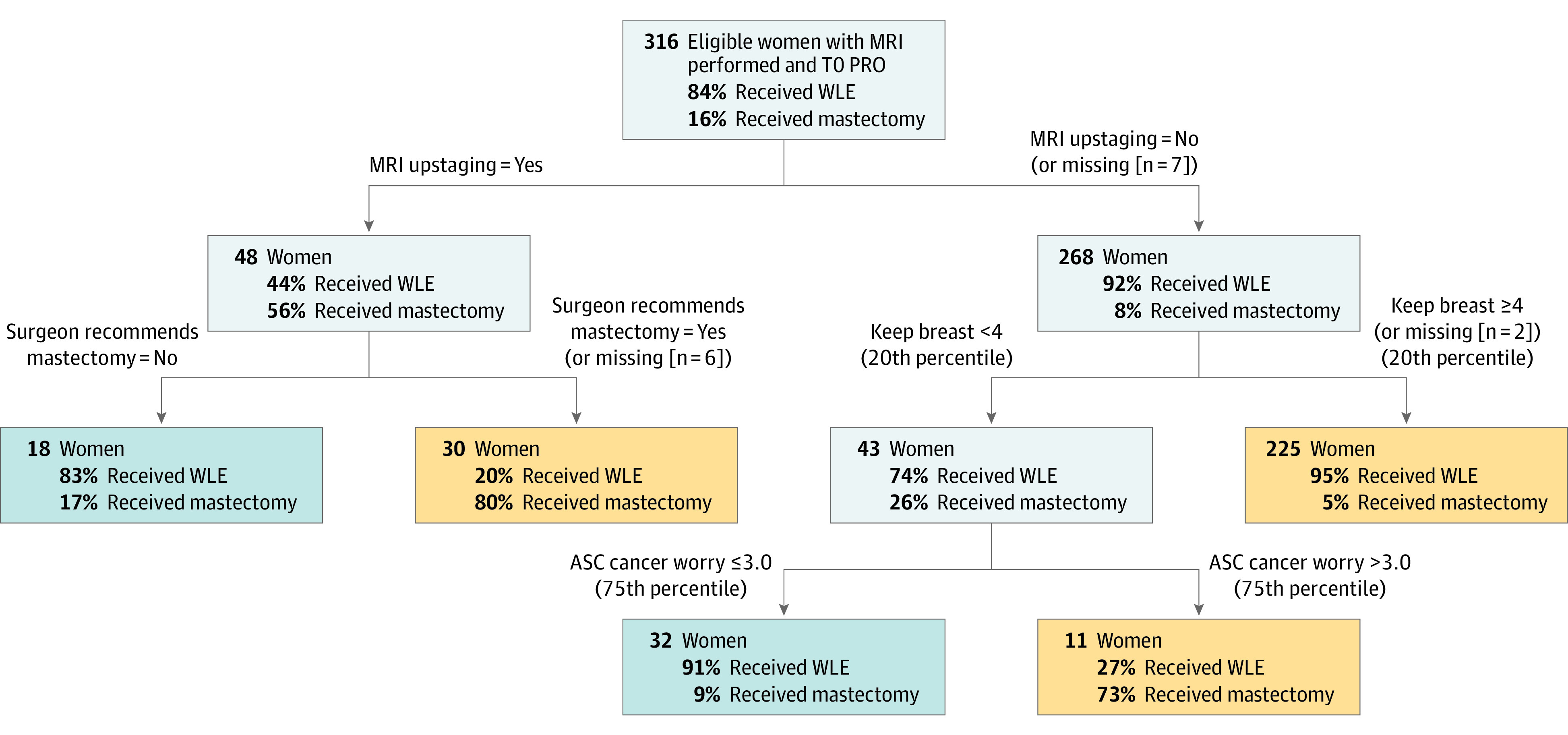
Classification Tree for Initial Surgery Received Using internal validation, the overall misclassification rate was 9% (29 of 316), with 97% (258 of 267) of women who initially received wide local excision (WLE) correctly classified, and 59% (29 of 49) of women who initially received mastectomy correctly classified. ASC indicates Assessment of Survivor Concerns; MRI, magnetic resonance imaging; PRO, patient-reported outcome; T0, time of registration.

The derived classification tree for initial surgery received is shown in Figure 3. The same covariates that were significant in the multivariable logistic regression model comprise the classification tree, with MRI upstaging exhibiting the largest variable importance measure. The classification tree suggests further insights. Among women without MRI upstaging, most who valued keeping their breast received WLE (214 of 225 [95%]), whereas among those for whom keeping the breast was not as important but who exhibited high levels of cancer worry, most received mastectomy (8 of 11 [73%]). In addition, among women for whom the MRI indicated upstaging and the surgeon recommended mastectomy, 80% (24 of 30) received mastectomy. Based on internal validation, the overall misclassification rate was 9% (29 of 316), with 97% (258 of 267) who initially received WLE and 59% (29 of 49) who initially received mastectomy correctly classified.

### Concordance Between T1 Surgery Preference and Surgery Received

eFigure 2 in the Supplement summarizes women’s preferred vs received surgery. Excluding 1 woman with missing data and 8 women who were still unsure of their surgery choice, 96% (231 of 241) of the women received the surgery that corresponded to their final preferred surgery choice at time point T1 (κ = 0.79; 95% CI, 0.66-0.91).

Of the 10 women who ultimately received surgery discordant with their T1 preference, 1 woman preferred mastectomy but received WLE; the MRI did not indicate upstaging and the surgeon did not recommend mastectomy. Nine women preferred WLE but received mastectomy; 6 had MRI upstaging, the surgeon recommended mastectomy in 2 additional women (although the MRI did not indicate upstaging), and for 1 woman, potential explanations were not evident.

### Sensitivity Analyses

The models were repeated after removing women whose ADI rankings were based on mailing to a post office box vs street address (n = 10) or only on the 5-digit zip code (n = 6). The classification tree was unaffected and similar point estimates were obtained for the logistic regression models.

## Discussion

Among women with DCIS, age, treatment goals, and concerns were associated with their surgical preference before MRI receipt. After MRI receipt and surgeon consultation, surgical preference was associated with MRI upstaging. Magnetic resonance imaging upstaging, surgeon recommendation, self-reported importance of keeping the breast, and cancer worry were associated with received surgery. A derived classification tree correctly classified the initial surgery for 91% of the women. Only 4% of women in the sample received a type of surgery that did not match their final surgery preference. The ADI was not associated with surgery preference or surgery received.

Women’s choice for early-stage breast cancer treatment is complex and appears to be influenced by clinicopathological, surgeon, and individual factors.^19^ In previous studies, individual values and belief factors statistically significantly influenced treatment decisions in patients with breast cancer.^19,20,21^ In a population-based study of surgical treatment decisions in women with DCIS,^8^ women who were greatly influenced by the desire to “get rid of the disease” or reduce the need for additional surgery were more likely to select mastectomy, whereas body image and sexuality concerns were associated with receipt of lumpectomy. However, in the population in this study, the relative importance of self-reported treatment goals and concerns decreased with respect to articulated preference as MRI information became available and patients continued to engage in the decision-making process.

Younger women were more likely to prefer mastectomy before MRI in our study. Many previous studies reported an increased likelihood of mastectomy in younger age groups.^20^ This preference may be due to higher self-estimated risk for a new primary or recurrent cancer over their lifetime and perception of mastectomy as a more definitive treatment.^22^ However, in our population, the initial association between age and preferred surgery was superseded by MRI results, suggesting the important role of preoperative MRI on surgery choice.

After MRI receipt and surgical consultation, MRI upstaging and surgeon recommendation were the key variables for initial surgery received. Compared with diagnostic mammogram and/or ultrasonography, MRI can more accurately delineate the extent of disease and therefore may considerably influence patient choice.^23^ Other studies have shown surgeon influence and recommendation to be key factors in treatment decisions.^24,25,26^ Most women who received a recommendation from a surgeon followed that recommendation, even though they perceived they were given a choice^8^; perceived treatment preference of their surgeon was the most important factor associated with their own treatment choice.^24^ Robust communication between patients and surgeons regarding clinical benefits and risks of available treatment options may aid decision optimization, informed by patient treatment goals and values. Surgeons’ perception of the role of MRI findings in guiding their treatment recommendations and use of MRI information to aid the decision-making process remain to be elucidated.

Most women in our sample received surgery matching their final preference. In the few patients who did not receive their preferred surgery, additional suspicious MRI findings prompting mastectomy was the potential cause of discordance. Magnetic resonance imaging can identify more extensive disease and mammographically unsuspected multifocal, multicentric, or bilateral cancer. However, limited specificity can result in unwarranted extended surgical resections or mastectomies.^23^ Bilimoria et al^27^ observed surgical management change in 23% of women after routine MRI for invasive breast cancer; however, this change resulted in additional benefit in less than half of the patients after matching radiologic and pathologic findings.

Reexcision rates for DCIS range from 22% to 41%, up to 3 times higher than in invasive breast cancer.^28^ Reexcision rates in our study reached 21%. Information on the contribution of MRI findings in avoiding repeat surgery is scant, limited by design heterogeneity and single institution or small samples when prospectively evaluated, with pooling of DCIS and invasive breast cancer data. A systematic review did not identify studies on DCIS meeting inclusion criteria.^29^ Single-institution nonrandomized studies suggest reduction in reexcision rates of up to 70%.^30,31^ A small randomized trial testing the clinical utility of MRI in DCIS showed a reduction in reoperation rates with a per-protocol analysis, but was underpowered to demonstrate a similar reduction under intent-to-treat analyses.^30^ The value of preoperative MRI in DCIS remains to be definitively assessed using a fully powered randomized clinical trial. Our data suggest that the value of MRI may lie in its contribution to surgery choice, evidenced by the change in patient preference before and after MRI and surgical consultation.

Breast cancer outcome disparities have been attributed to race, with Black women experiencing lower rates of survival despite similar rates of incidence of breast cancer.^32^ However, additional nuance regarding disparity sources is needed. A previous study noted that, when accounting for neighborhood socioeconomic status, Black women were more likely to adhere to endocrine therapy compared with White women.^33^ In women with metastatic breast cancer, neighborhood socioeconomic status rather than race was associated with the probability of receiving surgery.^34^ In the present study, ADI, which is a measure of neighborhood socioeconomic status, was not associated with either preferred or received surgery.

### Strengths and Limitations

Study strengths include a prospective design allowing assessment of temporal changes in patients’ surgery preferences and the inclusion of multiple sites, including community practices and academic centers. The study also has limitations. First, the sample size was relatively small compared with previous studies using population-based cancer registries; nevertheless, our findings are concordant. Second, our population was largely composed of White women. Thus, our results may not be generalizable to all races and may have underestimated the potential racial and ethnic differences in the treatment decision-making process. Third, we did not require a specific method or inclusion of specific content during participant-surgeon consultation, and this heterogeneity may have influenced participant knowledge. Fourth, not all instances in which MRI indicated more extensive disease were confirmed by subsequent biopsy and/or surgery; thus, not all changes in patient preference were based on histologic confirmation of MRI findings. A previous E4112 publication reported a positive predictive value of 26% (95% CI, 15%-40%) among patients whose MRI showed additional disease.^35^ Thus, surgical management change mediated by MRI findings may have benefited some, but not all, patients in E4112. However, the present analysis showed an association between MRI upstaging and patient preference, irrespective of subsequent biopsy result. Thus, although we cannot infer causation from an association, it is possible that MRI results may have some degree of influence on treatment choice regardless of accuracy. In addition, we were only able to validate our reported models using internal validation. Validating the models in external data sets would likely provide more generalizable and realistic estimates of discriminatory ability and the rate of misclassification.

## Conclusions

Surgery preference in DCIS is not fixed and appears to respond dynamically to additional diagnostic information conferred by MRI and surgeon recommendation. The findings of this study highlight the importance of ensuring adequate information and ongoing communication about the clinical significance of MRI findings and the benefits and risks of available treatment options. Although the benefit of MRI in avoiding reexcision remains to be elucidated in a population that largely continues to prefer and receive WLE, preoperative use may be of value in determining patient preferences for mastectomy vs breast conservation.

## Notes

Supplement. **eMethods.** Data Collection **eTable 1.** Univariable (Unadjusted) Firth Penalized Maximum Likelihood Logistic Regression Models for Surgery Preference at T0, Surgery Preference at T1, and Initial Surgery Received **eTable 2.** Multivariable Firth Penalized Maximum Likelihood Multinomial Logistic Regression Model for Surgery Preference at T0, Modeled as a Three-Category Outcome (WLE Versus Mastectomy Versus I Don’t Know) **eFigure 1.** Schedule of Patient-Reported Outcomes (PRO) Data Collection **eFigure 2.** Patient Surgery Preference at T0, Change in Patient Surgery Preference From T0 to T1, and Concordance Between Patient Surgery Preference and Initial Surgery Received
