# Supplementary material for: Association Between Surgery Preference and Receipt in Ductal Carcinoma In Situ After Breast Magnetic Resonance Imaging: An Ancillary Study of the ECOG-ACRIN Cancer Research Group (E4112)
Source: JAMA Netw Open. 2022 May 10;5(5):e2210331. doi: 10.1001/jamanetworkopen.2022.10331 (PMC9092204; doi:10.1001/jamanetworkopen.2022.10331)
Supplement: Supplement. — eMethods. Data Collection eTable 1. Univariable (Unadjusted) Firth Penalized Maximum Likelihood Logistic Regression Models for Surgery Preference at T0, Surgery Preference at T1, and Initial Surgery Received eTable 2. Multivariable Firth Penalized Maximum Likelihood Multinomial Logistic Regression Model for Surgery Preference at T0, Modeled as a Three-Category Outcome (WLE Versus Mastectomy Versus I Don’t Know) eFigure 1. Schedule of Patient-Reported Outcomes (PRO) Data Collection eFigure 2. Patient Surgery Preference at T0, Change in Patient Surgery Preference From T0 to T1, and Concordance Between Patient Surgery Preference and Initial Surgery Received [file jamanetwopen-e2210331-s001.pdf]

## Supplementary Online Content

Fazeli S, Snyder BS, Gareen IF, et al. Association between surgery preference and receipt in ductal carcinoma in situ after breast magnetic resonance imaging: an ancillary study of the ECOG-ACRIN Cancer Research Group (E4112). *JAMA Netw Open*. 2022;5(5):e2210331. doi:10.1001/jamanetworkopen.2022.10331

**eMethods.** Data Collection

**eTable 1.** Univariable (Unadjusted) Firth Penalized Maximum Likelihood Logistic Regression Models for Surgery Preference at T0, Surgery Preference at T1, and Initial Surgery Received

**eTable 2.** Multivariable Firth Penalized Maximum Likelihood Multinomial Logistic Regression Model for Surgery Preference at T0, Modeled as a Three-Category Outcome (WLE Versus Mastectomy Versus I Don't Know)

**eFigure 1.** Schedule of Patient-Reported Outcomes (PRO) Data Collection

**eFigure 2.** Patient Surgery Preference at T0, Change in Patient Surgery Preference From T0 to T1, and Concordance Between Patient Surgery Preference and Initial Surgery Received

This supplementary material has been provided by the authors to give readers additional information about their work.

© 2022 Fazeli S et al. *JAMA Network Open.*

## eMethods. Data Collection

Collection of PRO data was coordinated by the ECOG-ACRIN Outcomes and Economics Assessment Unit (OEAU) located at Brown University. At E4112 study registration, women were offered the option of completing questionnaires using a web-based application or by mail (paper-and-pencil). Questionnaire administration was triggered based on completion of study milestones marked by submission of E4112 study forms.

Patients electing web-based completion were invited to complete web-based questionnaires via an email prompt. We provided a study-specific toll-free telephone number to provide direct contact with the OEAU for patients who had questions or needed assistance. Patients who failed to complete web-based questionnaires within a specified number of days received follow-up emails and, if there was no response, OEAU attempted to contact these patients by telephone.

For patients preferring paper administration, mailed questionnaire packets included a letter introducing the study and the study-specific toll-free phone number for patients to reach the OEAU staff should they have questions or need assistance, together with pre-addressed, stamped envelopes for return mailing to the OEAU. If patients did not complete and return the questionnaires within 10 working days of the date of the mailing, the OEAU attempted to contact patients by telephone.

To obtain ADI rankings, participant addresses were linked to the 2020 US census data at the block group level. If the address lookup failed, then the full nine-digit zip code (zip+4) was used in combination with a census block group crosswalk provided by the University of Wisconsin. If the full nine-digit zip code was not available, then the standard five-digit zipcode was used, averaging over all ratings returned. A small number of ADI rankings were based on post office (PO) boxes or only the five-digit zip code.

© 2022 Fazeli S et al. *JAMA Network Open.*

**eTable 1 –** Univariable (unadjusted) Firth penalized maximum likelihood logistic regression models for surgery preference at T0, surgery preference at T1, and initial surgery received [a].

| Independent Variables | T0 Surgery preference | | T1 surgery preference | | Initial surgery received | |
|---|---|---|---|---|---|---|
| | **Unadjusted Odds Ratio (95% CI) [b]** | **p-value** | **Unadjusted Odds Ratio (95% CI) [b]** | **p-value** | **Unadjusted Odds Ratio (95% CI) [c]** | **p-value** |
| **Age** (continuous, per 5-year increment) | 0.65 (0.47 - 0.90) | **0.01 *** | 0.84 (0.67 - 1.05) | 0.12 | 0.84 (0.72 - 0.98) | **0.03 *** |
| **Race**: Non-white vs White | 2.24 (0.66 – 7.54) | 0.19 | 1.05 (0.35 - 3.13) | 0.94 | 1.17 (0.58 - 2.37) | 0.67 |
| **Insurance status**: Private vs Medicare/Medicaid/Other government insurance/Uninsured | 1.09 (0.26 - 4.59) | 0.90 | 0.78 (0.30 - 2.04) | 0.61 | 1.32 (0.62 - 2.85) | 0.47 |
| **ADI** (continuous, per 10-percentile increase) | 1.00 (0.78 - 1.28) | 0.99 | 0.94 (0.78 - 1.13) | 0.51 | 0.92 (0.81 - 1.05) | 0.22 |
| **Treatment goals and concerns** | | | | | | |
| How important is it to you to keep your breast? (continuous) | 0.64 (0.52 - 0.79) | **<0.001 ***** | 0.86 (0.76 - 0.98) | **0.03 *** | 0.82 (0.75 - 0.90) | **<0.001 ***** |
| How important is it to you to remove your entire breast to gain peace of mind? (continuous) | 1.36 (1.11 - 1.67) | **0.003 **** | 1.26 (1.09 - 1.46) | **0.002 **** | 1.17 (1.07 - 1.29) | **0.001 **** |
| How important is it to you to avoid having radiation? (continuous) | 1.03 (0.84 - 1.25) | 0.80 | 0.94 (0.82 - 1.08) | 0.41 | 0.98 (0.89 - 1.09) | 0.73 |
| How important is it that the type of surgery you have would not interfere with your sex life in the long term? (continuous) | 0.88 (0.74 - 1.04) | 0.12 | 0.96 (0.85 - 1.08) | 0.48 | 0.94 (0.87 - 1.02) | 0.16 |
| **ASC cancer worry subscale** (continuous) | 1.47 (0.74 – 2.89) | 0.27 | 1.87 (1.10 - 3.19) | **0.02 *** | 1.61 (1.13 - 2.31) | **0.009 **** |
| **MRI upstaging**: Yes vs No | -- | -- | 11.93 (4.57 - 31.14) | **<0.001 ***** | 15.91 (7.64 - 33.14) | **<0.001 ***** |
| **Surgeon recommended mastectomy**: Yes vs No | -- | -- | 4.41 (1.74 - 11.16) | **0.002 **** | 7.32 (3.68 – 14.53) | **<0.001 ***** |

[a] For the surgery preference models, women who were unsure of their preference were excluded (n=50 at T0, and n=8 at T1).

[b] Probability of mastectomy preference was modelled. Thus, an unadjusted odds ratio (OR) >1 indicates higher odds of preferring mastectomy compared to WLE. For categorical variables, the OR is interpreted in relation to the indicated reference level. For Age, the OR is interpreted per 5-year increase; for ADI, the OR is interpreted per 10-percentile increase; for PRO variables modeled as continuous covariates (Keep breast, Remove breast, Avoid radiation, Sex life, ASC cancer worry subscale), the OR is interpreted per 1-unit increase on the respective scale.

[c] Probability of mastectomy was modelled. Thus, an unadjusted odds ratio >1 indicates higher odds of the patient receiving mastectomy vs WLE. Odds ratios are interpreted as per footnote a.

WLE= Wide local excision; ADI=Area Deprivation Index; ASC=Assessment of Survivor Concerns; CI=Confidence interval

*** <0.05 ** <0.01 *** <0.001**

© 2022 Fazeli S et al. *JAMA Network Open.*

**eTable 2 –** Multivariable Firth penalized maximum likelihood multinomial logistic regression model for surgery preference at T0, modeled as a three-category outcome (WLE versus mastectomy versus I don't know).

| Independent Variables | T0 Surgery Preference | | |
|---|---|---|---|
| | **Surgery Preference [a]** | **Adjusted Odds Ratio (95% CI) [a]** | **p-value** |
| **Age** (continuous, per 5-year increment) | I don't know vs Mastectomy | 1.77 (1.15, 2.72) | **0.009 **** |
| | I don't know vs WLE | 1.04 (0.85, 1.29) | 0.68 |
| | Mastectomy vs WLE | 0.59 (0.39, 0.90) | **0.01 *** |
| **Race**: Non-white vs White | I don't know vs Mastectomy | 0.42 (0.08, 2.09) | 0.29 |
| | I don't know vs WLE | 1.44 (0.60, 3.46) | 0.41 |
| | Mastectomy vs WLE | 3.46 (0.72, 16.71) | 0.12 |
| **Insurance status:** Private vs Medicare/Medicaid/Other government insurance/Uninsured | I don't know vs Mastectomy | 2.22 (0.37, 13.46) | 0.39 |
| | I don't know vs WLE | 0.97 (0.37, 2.51) | 0.95 |
| | Mastectomy vs WLE | 0.44 (0.07, 2.57) | 0.36 |
| **ADI** (continuous, per 10-percentile increment) | I don't know vs Mastectomy | 1.14 (0.83, 1.58) | 0.42 |
| | I don't know vs WLE | 0.96 (0.82, 1.13) | 0.62 |
| | Mastectomy vs WLE | 0.84 (0.61, 1.16) | 0.29 |
| **Treatment goals and concerns** | | | |
| How important is it to you to keep your breast? (continuous) | I don't know vs Mastectomy | 1.19 (0.92, 1.53) | 0.19 |
| | I don't know vs WLE | 0.69 (0.61, 0.79) | **<0.001 ***** |
| | Mastectomy vs WLE | 0.59 (0.45, 0.76) | **<0.001 ***** |
| How important is it to you to remove your entire breast to gain peace of mind? (continuous) | I don't know vs Mastectomy | 0.87 (0.67, 1.12) | 0.27 |
| | I don't know vs WLE | 1.23 (1.10, 1.39) | **<0.001 ***** |
| | Mastectomy vs WLE | 1.43 (1.10, 1.84) | **0.006 **** |
| How important is it to you to avoid having radiation? (continuous) | I don't know vs Mastectomy | 0.92 (0.73, 1.17) | 0.50 |
| | I don't know vs WLE | 1.03 (0.91, 1.17) | 0.63 |
| | Mastectomy vs WLE | 1.12 (0.88, 1.41) | 0.35 |
| How important is it that the type of surgery you have would not interfere with your sex life in the long term? (continuous) | I don't know vs Mastectomy | 1.14 (0.91, 1.44) | 0.26 |
| | I don't know vs WLE | 1.05 (0.94, 1.18) | 0.36 |
| | Mastectomy vs WLE | 0.92 (0.73, 1.16) | 0.49 |
| **ASC cancer worry subscale** (continuous) | I don't know vs Mastectomy | 1.46 (0.57, 3.69) | 0.43 |
| | I don't know vs WLE | 1.41 (0.89, 2.23) | 0.14 |
| | Mastectomy vs WLE | 0.97 (0.39, 2.41) | 0.94 |

[a] Adjusted odds ratios are in reference to the rightmost category. For example, for Mastectomy vs WLE, an odds ratio (OR) >1 indicates higher odds of preferring mastectomy compared to WLE; for I don't know vs WLE, an OR >1 indicates higher odds of uncertainty compared to preferring WLE; for I don't know vs Mastectomy, an OR >1 indicates higher odds of uncertainty compared to preferring mastectomy. For categorical variables, the OR is interpreted in relation to the indicated reference level. For Age, the OR is interpreted per 5-year increase; for the ADI, the OR is interpreted per 10-percentile increase; for PRO variables modeled as continuous covariates (Keep breast, Remove breast, Avoid radiation, Sex life, ASC cancer worry subscale), the OR is interpreted per 1-unit increase on the respective scale.

WLE= Wide local excision; ADI=Area Deprivation Index; ASC=Assessment of Survivor Concerns; CI=Confidence Interval
*** <0.05 ** <0.01 *** <0.001**

© 2022 Fazeli S et al. *JAMA Network Open.*

**eFigure 1:** Schedule of patient-reported outcomes (PRO) data collection.

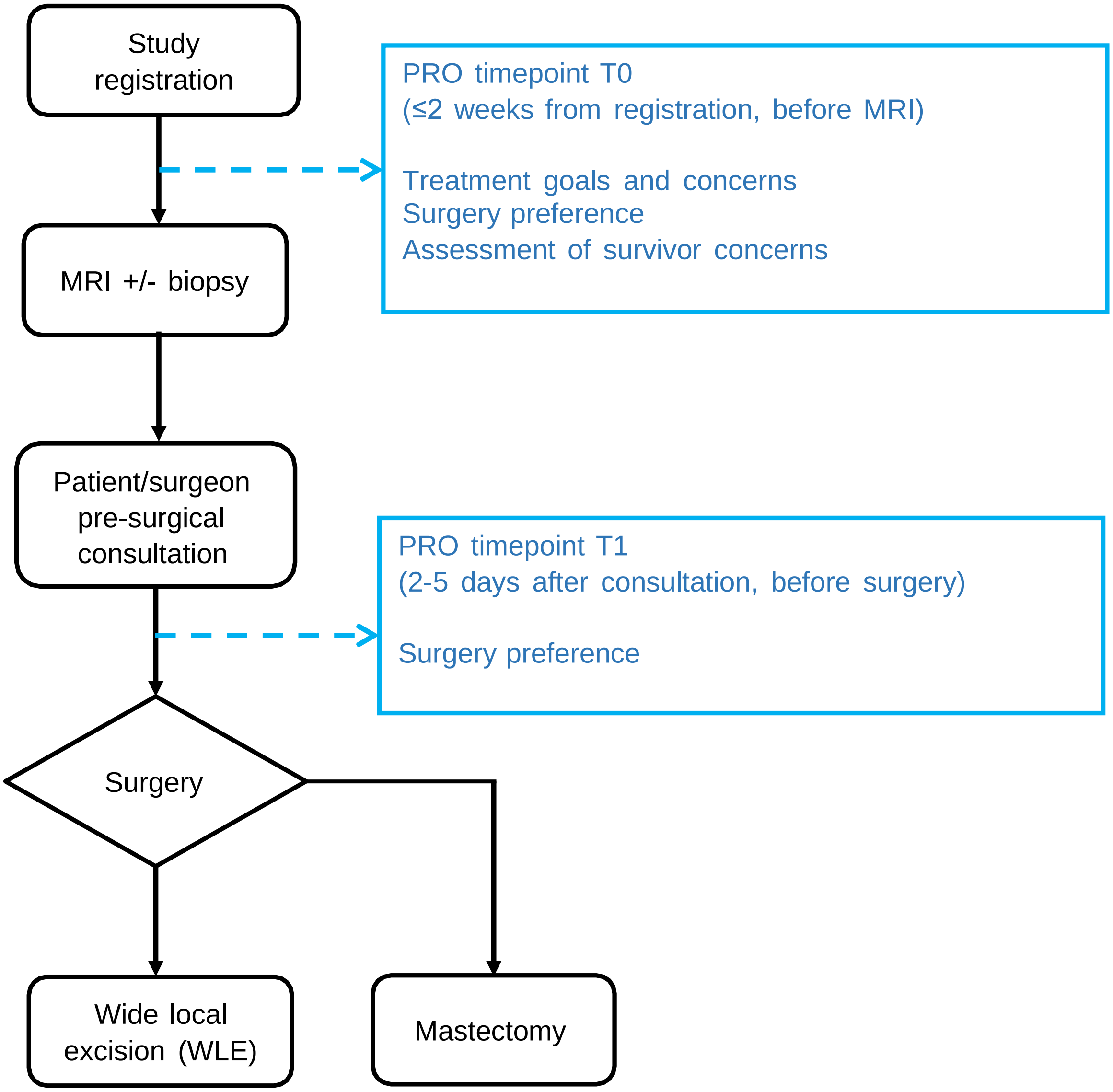


© 2022 Fazeli S et al. *JAMA Network Open.*

**eFigure 2**: Patient surgery preference at T0, change in patient surgery preference from T0 to T1, and concordance between patient surgery preference and initial surgery received.

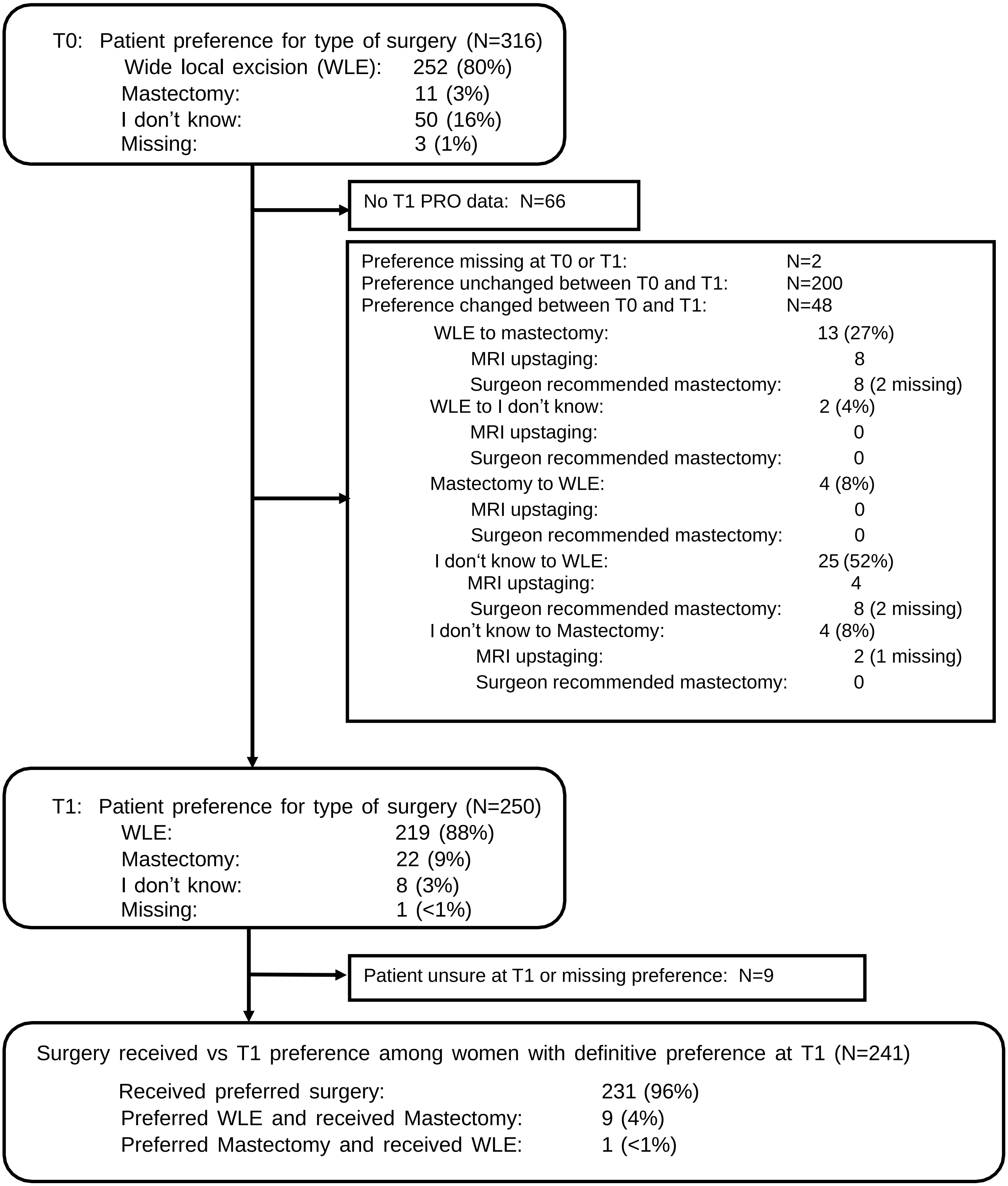


© 2022 Fazeli S et al. *JAMA Network Open.*
